# Functional clustering of time series gene expression data by Granger causality

**DOI:** 10.1186/1752-0509-6-137

**Published:** 2012-10-30

**Authors:** André Fujita, Patricia Severino, Kaname Kojima, João Ricardo Sato, Alexandre Galvão Patriota, Satoru Miyano

**Affiliations:** 1 Institute of Mathematics and Statistics, University of São Paulo, Rua do Matão, 1010, São Paulo 05508-090, Brazil; 2 Center for Experimental Research, Albert Einstein Research and Education Institute, Av. Albert Einstein, 627 - São Paulo, 05652-000, Brazil; 3 Human Genome Center, Institute of Medical Science, The University of Tokyo, 4-6-1 Shirokanedai, Minato-ku, Tokyo, 108-8639, Japan; 4 Center of Mathematics, Computation and Cognition, Universidade Federal do ABC, Rua santa Adélia, 166 - Santo André, 09210-170, Brazil

## Abstract

**Background:**

A common approach for time series gene expression data analysis includes the clustering of genes with similar expression patterns throughout time. Clustered gene expression profiles point to the joint contribution of groups of genes to a particular cellular process. However, since genes belong to intricate networks, other features, besides comparable expression patterns, should provide additional information for the identification of functionally similar genes.

**Results:**

In this study we perform gene clustering through the identification of Granger causality between and within sets of time series gene expression data. Granger causality is based on the idea that the cause of an event cannot come after its consequence.

**Conclusions:**

This kind of analysis can be used as a complementary approach for functional clustering, wherein genes would be clustered not solely based on their expression similarity but on their topological proximity built according to the intensity of Granger causality among them.

## Background

Gene network analysis of complex datasets, such as DNA microarray results, aims to identify relevant structures that help the understanding of a certain phenotype or condition. These networks comprise hundreds to thousands of genes that may interact generating intricate structures. Consequently, pinpointing genes or sets of genes that play a crucial role becomes a complicated task.

Common analyses explore gene-gene level relationships and generate broad networks. Although this is a valuable approach, genes might interact more intensely to a few members of the network, and the identification of these so-called sub-networks should lead to a better comprehension of the entire regulatory process.

**Figure 1:**
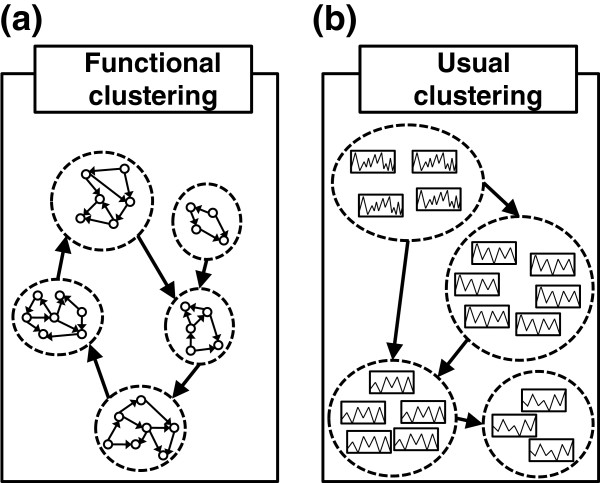
**Regulatory networks.(a)** Functional clustering. Genes are clustered based on their topological proximity given by Granger causality. **(b)** Usual clustering. Genes are clustered based on the similarity between gene expression levels.

Several *in silico* methodologies are available for the identification of sub-networks, or clusters, within a given dataset [1-5]. Most of the times, the identified clusters group genes based on similar patterns of expression in time. In a different manner, the identification of Granger causality [6] within a network allows the clustering of genes based on their topological proximity in the network [7,8]. Briefly, Granger causality [6] analysis identifies interaction in terms of temporal precedence (the cause comes before its effect) [6] and may generate a set of sub-networks within which Granger causality is intense among genes. As a result, genes are grouped depending on how close they are in terms of Granger causality. Figure 1a illustrates the clustering based on the network topological proximity while Figure 1b shows the clustering based on similar expression patterns. 

The concept of Granger causality [6] has been previously shown to help in the identification and interpretation of regulatory networks in time series gene expression datasets [9-18]. The main advantage of Granger causality analysis in the context of gene expression datasets consists in the fact that each edge of the network represents the information flow from one gene to another [19]. Nevertheless, it is necessary to point out that Granger causality is not effective causality in the Aristothelic sense because it is based on prediction and numerical calculations. Fujita *et al*. [20-22] suggested a concept for the identification of Granger causality between groups of time series. The application was, however, limited to scenarios when clusters could be previously defined based on particular data characteristics. Here, we propose a method to define clusters by their topological proximity in the network. For this purpose we introduce an extension of the concept of *functional clustering*, initially proposed by [23] in neuroscience. In [23], they applied mutual information in order to group the most active brain regions. We are interested in clustering the genes by using the concept of information flow [19] between *sets* of time series [20]. The gene expression time series are grouped depending on the hidden structure underlying the network topology, in a way that genes which are topologically close in terms of Granger causality are clustered (Figure 1a). We use the generalization of Granger causality for sets of time series datasets proposed by [20,21] in order to define concepts of distance, degree and flow useful to determine gene sets that highly interact in terms of Granger causality. In other words, we will derive the Granger causality-based functional clustering directly from the time series gene expression data. For this purpose, an approach that allows the identification of the optimum number of clusters for a given dataset is also presented.

## Materials and Methods

### Granger causality for sets of time series

Granger causality identification is a potential approach for the detection of possible interactions in a data driven framework couched in terms of temporal precedence. The main idea is that temporal precedence does not imply, but may help to identify causal relationships, since a cause never occurs after its effect.

A formal definition of Granger causality for sets of time series [20] can be given as follows.

#### Definition 1

[20]*Granger causality for sets of time series: Suppose thatℑ*_*t*_*is a set containing all relevant information available up to and including time-pointt*. *Let***X**_*t*_, $\left(X_{t}^{i}\right)$*and*$\left(X_{t}^{j}\right)$*be sets of time series containingp*, *mandntime series, respectively, where*$\left(X_{t}^{i}\right)$*and*$\left(X_{t}^{j}\right)$*are disjoint subsets of***X**_*t*_, *i.e., each time series only belongs to one set, and thus,p*≥*m* + *n*. *Let***X**_*t*_(*h*|*ℑ*_*t*_) *be the optimal (i.e., the one which produces the minimum mean squared error (MSE) prediction)h-step predictor of the set ofmtime series*$\left(X_{t}^{i}\right)$*from the time pointt*, *based on the information inℑ*_*t*_. *The forecast MSE of the linear combination of*$\left(X_{t}^{i}\right)$*will be denoted byΩ*_**X**_(*h*|*ℑ*_*t*_). *The set ofntime series*$\left(X_{t}^{j}\right)$*is said to Granger-cause the set ofmtime series*$\left(X_{t}^{i}\right)$*if*

(1) $$\Omega_{X}(h|{ℑ}_{t})\;<\;\Omega_{X}\;(h|{ℑ}_{t}\setminus \{X_{s}^{j}|s\leq t\})\;\text{for}\;\text{at}\;\text{least}\;\text{one}\;h\;=\;1,2,\ldots$$

*where*$\left({ℑ}_{t}\setminus \{X_{s}^{j}|s\leq t\}\right)$*is the set containing all relevant information except for the information in the past and present of*$\left(X_{t}^{j}\right)$. *In other words, if*$\left(X_{t}^{i}\right)$*can be predicted more accurately when the information in*$\left(X_{t}^{j}\right)$*is taken into account, then*$\left(X_{t}^{j}\right)$*is said to be Granger-causal for*$\left(X_{t}^{i}\right)$.

For the linear case, $\left(X_{t}^{j}\right)$ is Granger non-causal for $\left(X_{t}^{i}\right)$ if the following condition holds:

(2) $$\text{CCA}(X_{t}^{i},X_{t-1}^{j}|X_{t}\setminus \{X_{t-1}^{j}\})=\rho =0,$$

where *ρ* is the largest correlation calculated by Canonical Correlation Analysis (CCA).

In order to simplify both notation and concepts, only the identification of Granger causality for sets of time series in an Autoregressive process of order one is presented. Generalizations for higher orders are straightforward.

### Functional clustering in terms of Granger causality

There are numerous definitions for clusters in networks in the literature [24]. A functional cluster in terms of Granger causality can be defined as a subset of genes that strongly interact among themselves but interact weakly with the rest of the network.

A usual approach for network clustering when the structure of the graph is known is the spectral clustering proposed by [25]. However, in biological data, the structure of the regulatory network is usually unknown.

In order to overcome this limitation, we developed a framework to cluster genes by their topological proximity using the time series gene expression information. We developed concepts of distance and degree for sets of time series based on Granger causality, and combined them to the modified spectral clustering algorithm. The procedures are detailed below.

#### Functional clustering

Given a set of time series $\left(x_{t}^{1},x_{t}^{2},\ldots ,x_{t}^{p}\right)$ (where *p* is the number of time series) and a definition of similarity *w*_*ij*_ ≥ 0 between all pairs of data points $\left(x_{t}^{i}\right)$ and $\left(x_{t}^{j}\right)$, the intuitive goal of clustering is to divide the time series into several groups such that time series in the same group are highly connected by Granger causality and time series in different groups are not connected or show few connections to each other. One usual representation of the connectivity between time series is in the form of graph *G* = (*V*,*E*). Each vertex *v*_*i*_ in this graph represents a time series gene expression $\left(x_{t}^{i}\right)$. Two vertices are connected if the similarity *w*_*ij*_ between the corresponding time series $\left(x_{t}^{i}\right)$ and $\left(x_{t}^{j}\right)$ is not zero (the edge of the graph is weighted by *w*_*ij*_). In other words, a *w*_*ij*_ > 0 represents existence of Granger causality between time series $\left(x_{t}^{i}\right)$ and $\left(x_{t}^{j}\right)$ and *w*_*ij*_ = 0 represents Granger non-causality. The problem of clustering can now be reformulated using the similarity graph: we want to find a partition of the graph such that there is less Granger causality between different groups and more Granger causality within the group.

Let *G* = (*V*,*E*) be an undirected graph with vertex set *V* = {*v*_1_,…,*v*_*p*_}(where each vertex represents one time series) and weighted edges set *E*. In the following we assume that the graph *G* is weighted, that is each edge between two vertices *v*_*i*_ and *v*_*j*_ carries a non-negative weight *w*_*ij*_ ≥ 0. The weighted adjacency matrix of the graph is the matrix **W** = *w*_*ij*_; *i*,*j* = 1,…,*p*. If *w*_*ij*_ = 0, this means that the vertices *v*_*i*_ and *v*_*j*_ are not connected by an edge. As *G* is undirected, we require *w*_*ij*_ = *w*_*ji*_. Therefore, in terms of Granger causality, *w*_*ij*_ can be set as the distance between two time series $\left(x_{t}^{i}\right)$ and $\left(x_{t}^{j}\right)$. This distance can be defined as

##### Definition 2

*Distance between two (sets of) time series*$\left(x_{t}^{i}\right)$*and*$\left(x_{t}^{j}\right)$:

(3) $$\text{dist}\left(x_{t}^{i},x_{t}^{j}\right)=1-\frac{\left|\text{CCA}(x_{t}^{i},x_{t-1}^{j})|+|\text{CCA}(x_{t}^{j},x_{t-1}^{i})\right|}{2}.$$

Notice that $\left(\text{CCA}(x_{t}^{i},x_{t-1}^{j})\right)$ is the Granger causality from time series $\left(x_{t}^{j}\right)$ to $\left(x_{t}^{i}\right)$. In the case of sets of time series, just replace $\left(x_{t}^{i}\right)$ and $\left(x_{t}^{j}\right)$ by the set of time series $\left(X_{t}^{i}\right)$ and $\left(X_{t}^{j}\right)$[20,21]. Since absolute value of CCA ranges from zero to one and the higher the CCA, the higher is the quantity of information flow, it is possible to see that the higher the CCA, the shorter the distance is. Furthermore, it is necessary to point out that the average between $\left(\text{CCA}(x_{t}^{i},x_{t-1}^{j})\right)$ and $\left(\text{CCA}(x_{t}^{j},x_{t-1}^{i})\right)$ is calculated because the distance must be symmetric. The intuitive idea consists on the fact that the higher is the CCA coefficient, the lower is the distance between the time series (or sets of time series) independent of the direction of Granger causality.

Moreover, notice that the CCA is the Pearson correlation after dimension reduction, therefore, $\left(\text{dist}(x_{t}^{i},x_{t}^{j})\right)$ satisfies three out of four criteria for distances: (i) non-negativity; (ii) identity of indiscernible; and (iii) symmetry; and does not satisfy the (iv) triangular inequality, therefore, Pearson correlation is not a real metric. However, it is commonly used as a distance measure in several gene expression data analysis [26,27]. The main advantage with this definition of distance is the fact that it is possible to interpret the clustering process by a Granger causality concept.

Another necessary concept is the idea of degree of a time series $\left(x_{t}^{i}\right)$ (vertex *v*_*i*_) which can be defined as

##### Definition 3

*Degree of*$\left(x_{t}^{i}\right)$*is defined by:*

(4) $$\text{degree}(x_{t}^{i})=\frac{\text{in-degree}(x_{t}^{i})+\text{out-degree}(x_{t}^{i})}{2},$$

where in-degree and out-degree are respectively

(5) $$\text{in-degree}(x_{t}^{i})=|\text{CCA}(x_{t}^{i},X_{t-1}|X_{t}\setminus \{X_{t-1}\})|$$

(6) $$\text{out-degree}(x_{t}^{i})=|\text{CCA}(X_{t},x_{t-1}^{i}|X_{t}\setminus \{x_{t-1}^{i}\})|.$$

Notice that in-degree and out-degree represent the total information flow that “enters” and “leaves” the vertex *v*_*i*_, respectively. Therefore, the degree of vertex *v*_*i*_ contains the total information flow passing through vertex *v*_*i*_.

Without loss of generality, it is possible to extend the concept of degree of a vertex *v*_*i*_ (time series $\left(x_{t}^{i}\right)$) to a set of time series (sub-network) $\left(X_{t}^{u}\right)$, where *u* = 1,…,*k* and *k* is the number of sub-networks.

##### Definition 4

*Degree of sub-network*$\left(X_{t}^{u}\right)$*is defined by:*

(7) $$\text{degree}(X_{t}^{u})=\frac{\text{in-degree}(X_{t}^{u})+\text{out-degree}(X_{t}^{u})}{2},$$

where in-degree and out-degree are respectively

(8) $$\text{in-degree}(X_{t}^{u})=|\text{CCA}(X_{t}^{u},X_{t-1}|X_{t}\setminus \{X_{t-1}\})|,$$

(9) $$\text{out-degree}(X_{t}^{u})=|\text{CCA}(X_{t},X_{t-1}^{u}|X_{t}\setminus \{X_{t-1}^{u}\})|.$$

Now, by using the definitions of distance and degrees for time series and sets of time series in terms of Granger causality, it is possible to develop a spectral clustering-based algorithm to identify sub-networks (set of time series that are highly connected within sets and poorly connected between sets) in the regulatory networks. The algorithm based on spectral clustering [25] is as follows: 

**Input:** The *p* time series ( $\left(x_{t}^{i};i=1,\ldots ,p\right)$) and the number *k* of sub-networks to construct.

**Step 1:** Let **W** be the (*p* × *p*) symmetric weighted adjacency matrix where $\left(w_{i,j}=w_{j,i}=1-\text{dist}(x_{t}^{i};x_{t}^{j}),i,j=1,\ldots ,p\right)$.

**Step 2:** Compute the non-normalized (*p* × *p*) Laplacian matrix **L** as (Mohar, 1991)

(10) L=D−W

where **D** is the (*p* × *p*) diagonal matrix with the degrees *d*_1_,…,*d*_*p*_( $\left(\text{degree}(x_{t}^{i})=d_{i};i=1,\ldots ,p\right)$) on the diagonal.

**Step 3:** Compute the first *k* eigenvectors {**e**_1_,…,**e**_*k*_} (corresponding to the *k* largest eigenvalues) of **L**.

**Step 4:** Let **U** ∈ *ℜ*^*p*×*k*^ be the matrix containing the vectors {**e**_1_,…,**e**_*k*_} as columns.

**Step 5:** For *i* = 1,…,*p*, let **y**_*i*_ ∈ *ℜ*^*k*^ be the vector corresponding to the *i*th row of **U**.

**Step 6:** Cluster the points (**y**_*i*_)_*i*=1,…,*p*_ ∈ *ℜ*^*k*^ with the *k*-means algorithm into clusters {**X**_1_,…,**X**_*k*_}. For *k*-means, one may select a large number of initial values to achieve (or to be closer) the global optimum configuration. In our simulations, we generated 100 different initial values.

**Output:** Sub-networks {**X**_1_,…,**X**_*k*_}.

Notice that this clustering approach does not infer the entire structure of the network.

#### Estimation of the number of clusters

The method presented so far describes a framework for clustering genes (time series) using their topological proximity in terms of Granger causality.

Now, the challenge consists in determining the optimum number of sub-networks *k*. The choice of the number of sub-networks *k* is often difficult depending on what the researcher is interested in. In our specific problem, one is interested in identifying the clusters presenting dense connectivity within a cluster and sparse connectivity between clusters.

In order to determine the most appropriate number of clusters in this specific context, we used a variant of the silhouette method [28].

Let us first define the cluster index *s*(*i*) in the case of dissimilarities. Take any time series $\left(x_{t}^{i}\right)$ in the data set, and denote by **A** the sub-network to which it has been assigned. When sub-network **A** contains other time series apart from $\left(x_{t}^{i}\right)$, then we can compute: $\left(a(i)=\text{dist}(x_{t}^{i},A)\right)$, which is the average dissimilarity of $\left(x_{t}^{i}\right)$ to **A**. Let us now consider any sub-network **C** which is different from **A** and compute: $\left(\text{dist}(x_{t}^{i},C)\right)$ which is the dissimilarity of $\left(x_{t}^{i}\right)$ to **C**. After computing $\left(\text{dist}(x_{t}^{i},C)\right)$ for all sub-networks **C** ≠ **A**, we set the smallest of those numbers and denote it by $\left(b(i)=\mathrm{mi}n_{C\neq A}\text{dist}(x_{t}^{i},C)\right)$. The sub-network **B** for which this minimum value is attained (that is, $\left(\text{dist}(x_{t}^{i},B)=b(i))\right)$ we call the neighbor sub-network, or cluster of $\left(x_{t}^{i}\right)$. The neighbor cluster would be the second-best cluster for time series $\left(x_{t}^{i}\right)$. In other words, if $\left(x_{t}^{i}\right)$ could not belong to sub-network **A**, the best sub-network to belong to would be **B**. Therefore, *b*(*i*) is very useful to know the best alternative cluster for the time series in the network. Note that the construction of *b*(*i*) depends on the availability of other sub-networks apart from **A**, thus it is necessary to assume that there is more than one sub-network *k* within a given network [28].

After calculating *a*(*i*) and *b*(*i*), the cluster index *s*(*i*) can be obtained by combining them as follows:

(11) $$s(i)=\frac{b(i)-a(i)}{\max(a(i),b(i))}.$$

Indeed, from the above definition we easily see that −1 ≤ *s*(*i*) ≤ 1 for each time series $\left(x_{t}^{i}\right)$. Therefore, there are at least three cases to be analyzed, namely, when *s*(*i*) ≈ 1 or *s*(*i*) ≈ 0 or *s*(*i*) ≈ −1. For cluster index *s*(*i*) to be close to one we require *a*(*i*) ≪ *b*(*i*). As *a*(*i*) is a measure of how dissimilar *i* is to its own sub-network, a small value means it is well matched. Furthermore, a large *b*(*i*) implies that *i* is badly matched to its neighboring sub-network. Thus, a cluster index *s*(*i*) close to one means that the gene is appropriately clustered. If *s*(*i*) is close to negative one, then by the same logic we see that $\left(x_{t}^{i}\right)$ would be more appropriate if it was clustered in its neighboring sub-network. A cluster index *s*(*i*) near zero means that the gene is on the border of two sub-networks. In other words, the cluster index *s*(*i*) can be interpreted as the fitness of the time series $\left(x_{t}^{i}\right)$ to the assigned sub-network.

The average cluster index *s*(*i*) of a sub-network is a measure of how tightly grouped all the genes in the sub-network are. Thus, the average cluster index *s*(*i*) of the entire dataset is a measure of how appropriately the genes have been clustered in a topological point of view and in terms of Granger causality.

#### Estimation of the number of clusters in biological data

**Figure 2:**
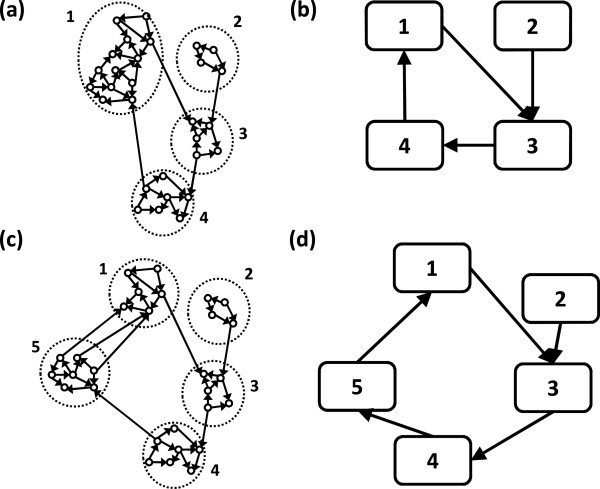
**(a) The representation of network with four clusters; (b) The network obtained by applying the proposed method with four clusters (*k*=4); (c) The representation of the network when the number of clusters is set to five; (d) The network obtained by applying the proposed method with five clusters (***k***=5).** The solid edges represent Granger causality. Notice that the structure of the “true” network (**(a)** and **(c)**) is not observed and it can only be estimated.

**Figure 3:**
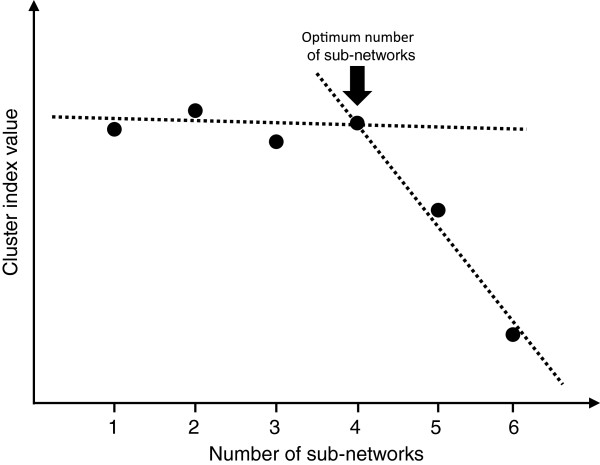
**The optimum number of sub-networks is indicated by the breakpoint in the graph.** The breakpoint appears when the number of sub-networks is greater than the adequate number of sub-networks. The breakpoint selection criterion is based on two linear regressions that best fit the data.

In order to estimate the most appropriate number of sub-networks present in the data set, we estimate the average cluster index *s* of the entire dataset for each number of clusters *k*. When the number of identified sub-networks is equal or lower than the adequate number of sub-networks, the cluster index values are very similar. However, when the number of identified sub-networks becomes higher than the adequate number of sub-networks, the cluster index value *s* decreases abruptly. This is due to the fact that one of the highly connected sub-networks is split into two new sub-networks. Notice that these two new sub-networks present high connectivity between them because they are in fact, only one sub-network. In order to illustrate this event, see Figure 2 for an example. In Figure 2a, genes in cluster 1 are highly interconnected. Now, suppose that one wants to increase the number of clusters by splitting cluster 1 into two clusters namely clusters 1 and 5 (Figure 2c). Notice that clusters 1 and 5 are highly connected between them. If the number of clusters is higher than the adequate number of clusters (four, in our case), the value *s* decreases substantially, since the Granger causality between clusters increases and the within cluster decreases. The breakpoint where the value *s* decreases abruptly can be used to determine the adequate number of sub-networks. In fact, this can be visually identified by analyzing the breakpoint at the plot similarly to the standard elbow method used in k-means. However, if one wants to determine the breakpoint in an objective manner, this can be done by adjusting two linear regressions, one with the first *q* dots and another with the remaining dots, thus identifying the breakpoint (the value *q*) that minimizes the sum of squared errors (Figure 3).

### Network construction

The network connecting clusters is constructed following procedures previously described [20,21]. Briefly, after Classification Expectation Maximization (CEM) [29] Principal Component Analysis (PCA) is used to remove redundancy and to extract the eigen-time series from each cluster. PCA allows us to keep only the most significant components leading to variability in the dataset, thus reducing the number of variables for subsequent processing. In this study, we retained only components accounting for more than 5% of the temporal variance in each cluster [22]. The eigen-time series are then clustered as described in the section Functional clustering and the network can be inferred by applying the method proposed by [20,21].

The Granger causality between cluster is identified by:

(12) $$\text{CCA}(X_{t}^{i},X_{t-1}^{j}|X_{t}\setminus \{X_{t-1}^{j}\})=\hat{\rho }\;\text{for all}\;i,j=1,\ldots ,k$$

where $\left(\hat{\rho }\right)$ is the sample canonical correlation between the sets $\left(X_{t}^{i}\right)$ and $\left(X_{t-1}^{j}\right)$ partialized by all information contained in **X**_*t*_ minus the set $\left(X_{t-1}^{j}\right)$.

Then, test

$\left({\text{H}}_{0}:\text{CCA}(X_{t}^{i},X_{t-1}^{j}|X_{t}\setminus \{X_{t-1}^{j}\})=\hat{\rho }=0\right)$ (Granger non-causality)

$\left({\text{H}}_{1}:\text{CCA}(X_{t}^{i},X_{t-1}^{j}|X_{t}\setminus \{X_{t-1}^{j}\})=\hat{\rho }\neq 0\right)$ (Granger causality) where H_0_ and H_1_ are the null and alternative hypothesis, respectively.

### Simulations

**Figure 4:**
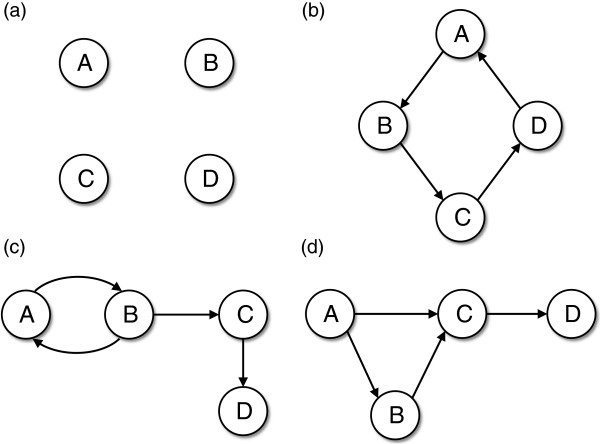
(a) Four independent sub-networks without Granger causality between them; (b) Four sub-networks in a cyclic graph; (c) Feedback loop between sub-networks A and B; (d) A network between sub-networks, where sub-networks A only sends Granger causality and D only receives Granger Causality.

Four sets of Monte Carlo simulations were carried out in order to evaluate the proposed approach under controlled conditions. The first scenario represents four sub-networks without Granger causality between them (Figure 4a). The second scenario consists of four sub-networks constituting a cyclic graph (Figure 4b). The third scenario presents a feedback loop between sub-networks A and B (Figure 4c). The fourth scenario is composed of a network with one sub-network (sub-network D) that only receives Granger causality and one sub-network (sub-network A) that only sends Granger causality (Figure 4d). Since biological data usually possess several highly correlated genes (genes which hold the same information from a statistical stand point), we constructed 10 highly correlated time series for each $\left(x_{t}^{i},i=1,\ldots ,20\right)$. In other words, $\left(x_{t}^{1}\right)$ is represented by 10 time series with correlation of 0.6 between them, $\left(x_{t}^{2}\right)$ is represented by 10 time series with correlation of 0.6 between them and so on. Therefore, instead of 20 time series, each scenario is in fact composed of 200 time series.

For each scenario, time series lengths varied: 50, 75, 1000 and 200 time points. The number of repetitions for each scenario is 1,000. The synthetic gene expression time series data in sub-networks A, B, C and D were generated by the following equations described below.

Simulation 1:

$$\left\{\begin{array}{ccc} \;x_{1,t}^{\text{A}} & = & \beta x_{1,t-1}^{\text{A}}-\beta x_{4,t-1}^{\text{A}}+\beta x_{3,t-1}^{\text{A}}+\varepsilon_{1,t}\\ \;x_{2,t}^{\text{A}} & = & \beta x_{1,t-1}^{\text{A}}-\beta x_{3,t-1}^{\text{A}}+\beta x_{4,t-1}^{\text{A}}+\varepsilon_{2,t}\\ \;x_{3,t}^{\text{A}} & = & \beta x_{1,t-1}^{\text{A}}-\beta x_{5,t-1}^{\text{A}}+\varepsilon_{3,t}\\ \;x_{4,t}^{\text{A}} & = & \beta x_{3,t-1}^{\text{A}}-\beta x_{5,t-1}^{\text{A}}+\varepsilon_{4,t}\\ \;x_{5,t}^{\text{A}} & = & \beta x_{2,t-1}^{\text{A}}-\beta x_{3,t-1}^{\text{A}}+\beta x_{4,t-1}^{\text{A}}+\varepsilon_{5,t}\\ \;x_{6,t}^{\text{B}} & = & \beta x_{9,t-1}^{\text{B}}-\beta x_{7,t-1}^{\text{B}}+\varepsilon_{6,t}\\ \;x_{7,t}^{\text{B}} & = & \beta x_{10,t-1}^{\text{B}}-\beta x_{7,t-1}^{\text{B}}+\varepsilon_{7,t}\\ \;x_{8,t}^{\text{B}} & = & \beta x_{10,t-1}^{\text{B}}-\beta x_{6,t-1}^{\text{B}}+\beta x_{7,t-1}^{\text{B}}+\varepsilon_{8,t}\\ \;x_{9,t}^{\text{B}} & = & \beta x_{7,t-1}^{\text{B}}-\beta x_{8,t-1}^{\text{B}}+\beta x_{9,t-1}^{\text{B}}-\beta x_{6,t-1}^{\text{B}}+\varepsilon_{9,t}\\ x_{10,t}^{\text{B}} & = & \beta x_{10,t-1}^{\text{B}}-\beta x_{6,t-1}^{\text{B}}+\beta x_{9,t-1}^{\text{B}}+\varepsilon_{10,t}\\ x_{11,t}^{\text{C}} & = & \beta x_{12,t-1}^{\text{C}}-\beta x_{15,t-1}^{\text{C}}+\varepsilon_{11,t}\\ x_{12,t}^{\text{C}} & = & \beta x_{14,t-1}^{\text{C}}-\beta x_{13,t-1}^{\text{C}}+\varepsilon_{12,t}\\ x_{13,t}^{\text{C}} & = & \beta x_{14,t-1}^{\text{C}}-\beta x_{11,t-1}^{\text{C}}+\varepsilon_{13,t}\\ x_{14,t}^{\text{C}} & = & \beta x_{13,t-1}^{\text{C}}-\beta x_{11,t-1}^{\text{C}}+\varepsilon_{14,t}\\ x_{15,t}^{\text{C}} & = & \beta x_{15,t-1}^{\text{C}}-\beta x_{12,t-1}^{\text{C}}+\beta x_{13,t-1}^{\text{C}}-\beta x_{14,t-1}^{\text{C}}+\varepsilon_{15,t}\\ x_{16,t}^{\text{D}} & = & \beta x_{19,t-1}^{\text{D}}-\beta x_{20,t-1}^{\text{D}}+\beta x_{17,t-1}^{\text{D}}-\beta x_{18,t-1}^{\text{D}}+\varepsilon_{16,t}\\ x_{17,t}^{\text{D}} & = & \beta x_{20,t-1}^{\text{D}}-\beta x_{17,t-1}^{\text{D}}+\beta x_{18,t-1}^{\text{D}}+\varepsilon_{17,t}\\ x_{18,t}^{\text{D}} & = & \beta x_{20,t-1}^{\text{D}}-\beta x_{18,t-1}^{\text{D}}+\varepsilon_{18,t}\\ x_{19,t}^{\text{D}} & = & \beta x_{17,t-1}^{\text{D}}-\beta x_{18,t-1}^{\text{D}}+\varepsilon_{19,t}\\ x_{20,t}^{\text{D}} & = & \beta x_{20,t-1}^{\text{D}}-\beta x_{19,t-1}^{\text{D}}+\varepsilon_{20,t} \end{array}\right)$$

Simulation 2:

$$\left\{\begin{array}{ccc} \;x_{1,t}^{\text{A}} & = & \beta x_{1,t-1}^{\text{A}}-\beta x_{4,t-1}^{\text{A}}+\beta x_{3,t-1}^{\text{A}}+\varepsilon_{1,t}\\ \;x_{2,t}^{\text{A}} & = & \beta x_{1,t-1}^{\text{A}}-\beta x_{3,t-1}^{\text{A}}+\beta x_{4,t-1}^{\text{A}}+\varepsilon_{2,t}\\ \;x_{3,t}^{\text{A}} & = & \beta x_{1,t-1}^{\text{A}}-\beta x_{5,t-1}^{\text{A}}+\gamma x_{19,t-1}^{\text{D}}+\varepsilon_{3,t}\\ \;x_{4,t}^{\text{A}} & = & \beta x_{3,t-1}^{\text{A}}-\beta x_{5,t-1}^{\text{A}}+\varepsilon_{4,t}\\ \;x_{5,t}^{\text{A}} & = & \beta x_{2,t-1}^{\text{A}}-\beta x_{3,t-1}^{\text{A}}+\beta x_{4,t-1}^{\text{A}}+\varepsilon_{5,t}\\ \;x_{6,t}^{\text{B}} & = & \beta x_{9,t-1}^{\text{B}}-\beta x_{7,t-1}^{\text{B}}+\gamma x_{3,t-1}^{\text{A}}+\varepsilon_{6,t}\\ \;x_{7,t}^{\text{B}} & = & \beta x_{10,t-1}^{\text{B}}-\beta x_{7,t-1}^{\text{B}}+\varepsilon_{7,t}\\ \;x_{8,t}^{\text{B}} & = & \beta x_{10,t-1}^{\text{B}}-\beta x_{6,t-1}^{\text{B}}+\beta x_{7,t-1}^{\text{B}}+\varepsilon_{8,t}\\ \;x_{9,t}^{\text{B}} & = & \beta x_{7,t-1}^{\text{B}}-\beta x_{8,t-1}^{\text{B}}+\beta x_{9,t-1}^{\text{B}}-\beta x_{6,t-1}^{\text{B}}+\varepsilon_{9,t}\\ x_{10,t}^{\text{B}} & = & \beta x_{10,t-1}^{\text{B}}-\beta x_{6,t-1}^{\text{B}}+\beta x_{9,t-1}^{\text{B}}+\varepsilon_{10,t}\\ x_{11,t}^{\text{C}} & = & \beta x_{12,t-1}^{\text{C}}-\beta x_{15,t-1}^{\text{C}}+\varepsilon_{11,t}\\ x_{12,t}^{\text{C}} & = & \beta x_{14,t-1}^{\text{C}}-\beta x_{13,t-1}^{\text{C}}+\varepsilon_{12,t}\\ x_{13,t}^{\text{C}} & = & \beta x_{14,t-1}^{\text{C}}-\beta x_{11,t-1}^{\text{C}}+\varepsilon_{13,t}\\ x_{14,t}^{\text{C}} & = & \beta x_{13,t-1}^{\text{C}}-\beta x_{11,t-1}^{\text{C}}+\gamma x_{10,t-1}^{\text{B}}+\varepsilon_{14,t}\\ x_{15,t}^{\text{C}} & = & \beta x_{15,t-1}^{\text{C}}-\beta x_{12,t-1}^{\text{C}}+\beta x_{13,t-1}^{\text{C}}-\beta x_{14,t-1}^{\text{C}}+\varepsilon_{15,t}\\ x_{16,t}^{\text{D}} & = & \beta x_{19,t-1}^{\text{D}}-\beta x_{20,t-1}^{\text{D}}+\beta x_{17,t-1}^{\text{D}}-\beta x_{18,t-1}^{\text{D}}+\varepsilon_{16,t}\\ x_{17,t}^{\text{D}} & = & \beta x_{20,t-1}^{\text{D}}-\beta x_{17,t-1}^{\text{D}}+\beta x_{18,t-1}^{\text{D}}+\varepsilon_{17,t}\\ x_{18,t}^{\text{D}} & = & \beta x_{20,t-1}^{\text{D}}-\beta x_{18,t-1}^{\text{D}}+\varepsilon_{18,t}\\ x_{19,t}^{\text{D}} & = & \beta x_{17,t-1}^{\text{D}}-\beta x_{18,t-1}^{\text{D}}+\varepsilon_{19,t}\\ x_{20,t}^{\text{D}} & = & \beta x_{20,t-1}^{\text{D}}-\beta x_{19,t-1}^{\text{D}}+\gamma x_{15,t-1}^{\text{C}}+\varepsilon_{20,t} \end{array}\right)$$

Simulation 3:

$$\left\{\begin{array}{ccc} \;x_{1,t}^{\text{A}} & = & \beta x_{1,t-1}^{\text{A}}-\beta x_{4,t-1}^{\text{A}}+\beta x_{3,t-1}^{\text{A}}+\varepsilon_{1,t}\\ \;x_{2,t}^{\text{A}} & = & \beta x_{1,t-1}^{\text{A}}-\beta x_{3,t-1}^{\text{A}}+\beta x_{4,t-1}^{\text{A}}+\varepsilon_{2,t}\\ \;x_{3,t}^{\text{A}} & = & \beta x_{1,t-1}^{\text{A}}-\beta x_{5,t-1}^{\text{A}}+\gamma x_{6,t-1}^{\text{B}}+\varepsilon_{3,t}\\ \;x_{4,t}^{\text{A}} & = & \beta x_{3,t-1}^{\text{A}}-\beta x_{5,t-1}^{\text{A}}+\varepsilon_{4,t}\\ \;x_{5,t}^{\text{A}} & = & \beta x_{2,t-1}^{\text{A}}-\beta x_{3,t-1}^{\text{A}}+\beta x_{4,t-1}^{\text{A}}+\varepsilon_{5,t}\\ \;x_{6,t}^{\text{B}} & = & \beta x_{9,t-1}^{\text{B}}-\beta x_{7,t-1}^{\text{B}}+\varepsilon_{6,t}\\ \;x_{7,t}^{\text{B}} & = & \beta x_{10,t-1}^{\text{B}}-\beta x_{7,t-1}^{\text{B}}+\gamma x_{3,t-1}^{\text{A}}+\varepsilon_{7,t}\\ \;x_{8,t}^{\text{B}} & = & \beta x_{10,t-1}^{\text{B}}-\beta x_{6,t-1}^{\text{B}}+\beta x_{7,t-1}^{\text{B}}+\varepsilon_{8,t}\\ \;x_{9,t}^{\text{B}} & = & \beta x_{7,t-1}^{\text{B}}-\beta x_{8,t-1}^{\text{B}}+\beta x_{9,t-1}^{\text{B}}-\beta x_{6,t-1}^{\text{B}}+\varepsilon_{9,t}\\ x_{10,t}^{\text{B}} & = & \beta x_{10,t-1}^{\text{B}}-\beta x_{6,t-1}^{\text{B}}+\beta x_{9,t-1}^{\text{B}}+\varepsilon_{10,t}\\ x_{11,t}^{\text{C}} & = & \beta x_{12,t-1}^{\text{C}}-\beta x_{15,t-1}^{\text{C}}+\varepsilon_{11,t}\\ x_{12,t}^{\text{C}} & = & \beta x_{14,t-1}^{\text{C}}-\beta x_{13,t-1}^{\text{C}}+\varepsilon_{12,t}\\ x_{13,t}^{\text{C}} & = & \beta x_{14,t-1}^{\text{C}}-\beta x_{11,t-1}^{\text{C}}+\varepsilon_{13,t}\\ x_{14,t}^{\text{C}} & = & \beta x_{13,t-1}^{\text{C}}-\beta x_{11,t-1}^{\text{C}}+\gamma x_{10,t-1}^{\text{B}}+\varepsilon_{14,t}\\ x_{15,t}^{\text{C}} & = & \beta x_{15,t-1}^{\text{C}}-\beta x_{12,t-1}^{\text{C}}+\beta x_{13,t-1}^{\text{C}}-\beta x_{14,t-1}^{\text{C}}+\varepsilon_{15,t}\\ x_{16,t}^{\text{D}} & = & \beta x_{19,t-1}^{\text{D}}-\beta x_{20,t-1}^{\text{D}}+\beta x_{17,t-1}^{\text{D}}-\beta x_{18,t-1}^{\text{D}}+\varepsilon_{16,t}\\ x_{17,t}^{\text{D}} & = & \beta x_{20,t-1}^{\text{D}}-\beta x_{17,t-1}^{\text{D}}+\beta x_{18,t-1}^{\text{D}}+\varepsilon_{17,t}\\ x_{18,t}^{\text{D}} & = & \beta x_{20,t-1}^{\text{D}}-\beta x_{18,t-1}^{\text{D}}+\varepsilon_{18,t}\\ x_{19,t}^{\text{D}} & = & \beta x_{17,t-1}^{\text{D}}-\beta x_{18,t-1}^{\text{D}}+\varepsilon_{19,t}\\ x_{20,t}^{\text{D}} & = & \beta x_{20,t-1}^{\text{D}}-\beta x_{19,t-1}^{\text{D}}+\gamma x_{15,t-1}^{\text{C}}+\varepsilon_{20,t} \end{array}\right)$$

Simulation 4:

$$\left\{\begin{array}{ccc} \;x_{1,t}^{\text{A}} & = & \beta x_{1,t-1}^{\text{A}}-\beta x_{4,t-1}^{\text{A}}+\beta x_{3,t-1}^{\text{A}}+\varepsilon_{1,t}\\ \;x_{2,t}^{\text{A}} & = & \beta x_{1,t-1}^{\text{A}}-\beta x_{3,t-1}^{\text{A}}+\beta x_{4,t-1}^{\text{A}}+\varepsilon_{2,t}\\ \;x_{3,t}^{\text{A}} & = & \beta x_{1,t-1}^{\text{A}}-\beta x_{5,t-1}^{\text{A}}+\varepsilon_{3,t}\\ \;x_{4,t}^{\text{A}} & = & \beta x_{3,t-1}^{\text{A}}-\beta x_{5,t-1}^{\text{A}}+\varepsilon_{4,t}\\ \;x_{5,t}^{\text{A}} & = & \beta x_{2,t-1}^{\text{A}}-\beta x_{3,t-1}^{\text{A}}+\beta x_{4,t-1}^{\text{A}}+\varepsilon_{5,t}\\ \;x_{6,t}^{\text{B}} & = & \beta x_{9,t-1}^{\text{B}}-\beta x_{7,t-1}^{\text{B}}+\varepsilon_{6,t}\\ \;x_{7,t}^{\text{B}} & = & \beta x_{10,t-1}^{\text{B}}-\beta x_{7,t-1}^{\text{B}}+\gamma x_{3,t-1}^{\text{A}}+\varepsilon_{7,t}\\ \;x_{8,t}^{\text{B}} & = & \beta x_{10,t-1}^{\text{B}}-\beta x_{6,t-1}^{\text{B}}+\beta x_{7,t-1}^{\text{B}}+\varepsilon_{8,t}\\ \;x_{9,t}^{\text{B}} & = & \beta x_{7,t-1}^{\text{B}}-\beta x_{8,t-1}^{\text{B}}+\beta x_{9,t-1}^{\text{B}}-\beta x_{6,t-1}^{\text{B}}+\varepsilon_{9,t}\\ x_{10,t}^{\text{B}} & = & \beta x_{10,t-1}^{\text{B}}-\beta x_{6,t-1}^{\text{B}}+\beta x_{9,t-1}^{\text{B}}+\varepsilon_{10,t}\\ x_{11,t}^{\text{C}} & = & \beta x_{12,t-1}^{\text{C}}-\beta x_{15,t-1}^{\text{C}}+\varepsilon_{11,t}\\ x_{12,t}^{\text{C}} & = & \beta x_{14,t-1}^{\text{C}}-\beta x_{13,t-1}^{\text{C}}+\varepsilon_{12,t}\\ x_{13,t}^{\text{C}} & = & \beta x_{14,t-1}^{\text{C}}-\beta x_{11,t-1}^{\text{C}}+\gamma x_{8,t-1}^{\text{B}}+\varepsilon_{13,t}\\ x_{14,t}^{\text{C}} & = & \beta x_{13,t-1}^{\text{C}}-\beta x_{11,t-1}^{\text{C}}+\gamma_{5,t-1}^{\text{A}}+\varepsilon_{14,t}\\ x_{15,t}^{\text{C}} & = & \beta x_{15,t-1}^{\text{C}}-\beta x_{12,t-1}^{\text{C}}+\beta x_{13,t-1}^{\text{C}}-\beta x_{14,t-1}^{\text{C}}+\varepsilon_{15,t}\\ x_{16,t}^{\text{D}} & = & \beta x_{19,t-1}^{\text{D}}-\beta x_{20,t-1}^{\text{D}}+\beta x_{17,t-1}^{\text{D}}-\beta x_{18,t-1}^{\text{D}}+\varepsilon_{16,t}\\ x_{17,t}^{\text{D}} & = & \beta x_{20,t-1}^{\text{D}}-\beta x_{17,t-1}^{\text{D}}+\beta x_{18,t-1}^{\text{D}}+\varepsilon_{17,t}\\ x_{18,t}^{\text{D}} & = & \beta x_{20,t-1}^{\text{D}}-\beta x_{18,t-1}^{\text{D}}+\varepsilon_{18,t}\\ x_{19,t}^{\text{D}} & = & \beta x_{17,t-1}^{\text{D}}-\beta x_{18,t-1}^{\text{D}}+\varepsilon_{19,t}\\ x_{20,t}^{\text{D}} & = & \beta x_{20,t-1}^{\text{D}}-\beta x_{19,t-1}^{\text{D}}+\gamma x_{15,t-1}^{\text{C}}+\varepsilon_{20,t} \end{array}\right)$$

where *β* = 0.6, *γ* = 0.3, *ε*_*i*,*t*_∼*N*(0,**Σ**) with

(13) $$\Sigma =I_{\left(20\times 20\right)}⊗\Gamma$$

and

(14) $$\Gamma ={\left(\begin{array}{c} \;\;1\;0.6\;\;\ldots \;\;0.6\\ \;0.6\;\;\;\;1\;\ddots \;\vdots \\ \;\vdots \;\;\;\ddots \;\ddots \;0.6\\ \;0.6\;\ldots \;\;0.6\;1 \end{array},\;\right)}_{\left(10\times 10\right)}$$

for *i* = 1,…,20.

### Actual biological data

In order to illustrate an application of the proposed approach, a dataset collected by [30] was used. The work presents whole genome gene expression data during the cell division cycle of a human cancer cell line (HeLa) characterized using cDNA microarrays. The dataset contains three complete cell cycles of ∼16 hours each, with a total of 48 time points distributed at intervals of one hour. The full dataset is available at: http://genome-www.stanford.edu/Human-CellCycle/HeLa/.

In order to evaluate our proposed approach, we chose to analyze the same gene set examined in Figure 5 of [10], which comprised a set of 50 genes. 

**Figure 5:**
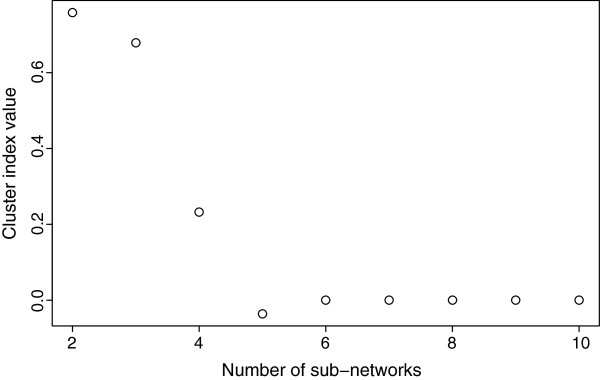
The optimum number of sub-networks in the actual biological data is indicated by the breakpoint in the graph (the optimum number in this case is three).

## Results

### Simulated data

In order to study the properties of the proposed functional clustering method and to check its consistency, we performed four simulations with distinct network characteristics in terms of structure and Granger causality.

Table 1 describes the frequency that each number of clusters was identified as optimal in each simulation and time series length. Notice that the accuracy of the method in identifying the correct number of clusters clearly converges to 100% as the time series length increases (the correct number of clusters is four for all the scenarios). The same result was obtained with varying numbers of sub-networks or when Granger causality within clusters increased, demonstrating the consistency of the method. Moreover, both the cluster indices value and the respective standard deviation for each simulation and time series length are described. The average cluster index value was calculated by using the value at the breakpoint as described in Figure 3 in 1,000 repetitions. By analyzing Table 1, it is possible to verify that the longer the time series length, the smaller are the standard deviations and the greater is the silhouette width demonstrating that the method is consistent.

**Table 1:** Frequency of the selected number of clusters for each scenario and time series length

**Time series length/Number of clusters**	**1**	**2**	**3**	**4**		**6**	**5**	**silhouette width**
Scenario 1							
50	0	0	48	**700**	252	0	0.502 (0.098)
75	0	0	1	**785**	214	0	0.582 (0.054)
100	0	0	3	**805**	192	0	0.610 (0.042)
200	0	0	4	**825**	171	0	0.641 (0.034)
Scenario 2							
50	0	0	65	**713**	222	0	0.479 (0.112)
75	0	0	28	**760**	212	0	0.555 (0.071)
100	0	0	9	**834**	157	0	0.587 (0.050)
200	0	0	3	**883**	114	0	0.621 (0.029)
Scenario 3							
50	0	0	63	**666**	271	0	0.461 (0.123)
75	0	0	18	**784**	198	0	0.552 (0.078)
100	0	0	8	**851**	141	0	0.586 (0.050)
200	0	0	6	**883**	111	0	0.618 (0.031)
Scenario 4							
50	0	0	53	**686**	261	0	0.465 (0.110)
75	0	0	17	**786**	197	0	0.551 (0.075)
100	0	0	11	**815**	174	0	0.581 (0.055)
200	0	0	6	**887**	107	0	0.619 (0.033)

In bold are the correct number of clusters. Between brackets is one standard deviation for the silhouette width calculated in the breakpoint. For each scenario and each time series length, the number of repetitions was set to 1,000.

Table 2 describes the average of the frequency (in percentage) the time series were correctly clustered for each scenario and each time series length given the correct number of clusters. It is important to point out that the number of correctly classified time series increases as the time series length increases.

**Table 2:** Average of the percentage of correctly clustered time-series in 1,000 repetitions given the correct number of clusters

**Scenario/Time series length**	**50**	**75**	**100**	**200**
1	78.8	96.0	98.9	99.9
2	72.9	91.2	95.8	99.2
3	71.6	90.6	95.2	99.7
4	68.9	88.7	93.7	99.1

Table 3 represents the frequency (in percentage) each edge of the simulated network was identified when the estimated number of clusters were correctly identified as four. The correctly identified edges are in bold. Since the p-value threshold was set to 0.05, it is expected to identify ≈5*%* of false positive edges where there is indeed no Granger causality. In fact, where there is no Granger causality, the rate of false positives was controlled to 5%, and where there is Granger causality, the number of identified edges is clearly higher than where there is no Granger causality.

**Table 3:** Percentage of edges with time series length equals to 50/75/100/200 when the estimated number of clusters were correctly identified as four

**from/to**	**A**	**B**	**C**	**D**
Scenario 1				
A	**100/100/100/100**	6.7/6.3/5.2/5.4	8.9/6.0/5.0/5.3	4.8/5.7/5.4/4.5
B	6.9/7.1/5.5/6.8	**99.9/100/100/100**	7.8/6.2/6.3/4.6	5.6/6.9/4.9/5.6
C	7.6/5.9/6.5/5.6	6.9/7.7/4.7/5.1	**100/100/100/100**	4.9/5.4/5.7/5.8
D	6.2/5.3/5.1/4.7	5.3/5.2/5.3/5.7	7.0/5.2/5.2/5.6	**100/100/100/100**
Scenario 2				
A	**100/100/100/100**	**28.9/59.8/80.4/99.7**	8.0/6.4/6.8/5.2	6.4/6.6/5.0/5.0
B	5.4/5.3/5.5/4.6	**100/100/100/100**	**29.6/60.9/82.1/99.9**	6.4/6.3/5.7/5.7
C	7.5/5.4/6.7/4.5	8.8/6.6/6.6/6.3	**100/100/100/100**	**23.0/50.4/71.2/99.1**
D	**17.6/35.5/51.2/95.4**	6.5/4.2/3.4/5.0	12.5/10.4/7.5/5.0	**100/100/100/100**
Scenario 3				
A	**100/100/100/100**	**29.6/61.9/82.1/100**	7.8/7.3/4.5/5.0	7.4/6.8/4.6/5.2
B	**28.5/53.0/78.0/99.9**	**100/100/100/100**	**31.8/61.1/82.9/99.9**	7.0/7.1/6.2/4.7
C	8.4/6.9/6.4/5.6	7.6/7.8/7.3/5.2	**99.9/100/100/100**	**25.5/46.8/70.6/99.3**
D	6.8/5.6/5.8/5.0	5.5/4.5/5.7/4.3	13.9/8.2/6.1/5.4	**100/100/100/100**
Scenario 4				
A	**100/100/100/100**	**25.1/52.6/75.8/99.6**	**22.9/41.8/59.5/96.0**	6.8/5.8/5.2/4.7
B	6.7/5.9/5.7/5.9	**100/100/100/100**	**28.6/58.4/81.9/100**	7.9/6.0/6.1/5.2
C	9.3/8.8/6.1/6.2	8.8/6.2/6.3/4.5	**100/100/100/100**	**26.5/53.2/75.4/99.2**
D	5.4/5.8/5.1/4.7	5.8/5.0/4.2/5.2	14.9/11.9/7.9/5.4	**100/100/100/100**

The rows and columns represent the clusters A, B, C, and D. The rate of false positives was controlled to 5% (p-value < 0.05). The edges which actually exists in the network are shown in bold.

### Biological data

By applying the method described in section Functional clustering to the biological dataset, the optimum number of sub-networks was identified as three. Notice in Figure 5 that there is a clear breakpoint when the number of clusters is three.

**Figure 6:**
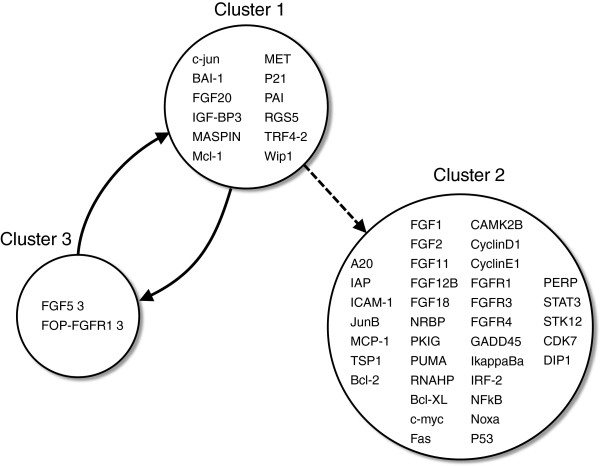
**The network obtained with three (*k*=3) sub-networks.** Solid arrows are significant Granger causality with p-value < 0.05 and dashed arrow is significant Granger causality with p-value < s0.10. The circles represent the clusters.

Once clusters were obtained, the cluster-cluster network (Figure 6) was modeled by applying the method described in [20,21]. Two of the depicted clusters, clusters one and two, provide interesting material for biological interpretation. Genes belonging to cluster two highlight expected interconnections in cell cycle regulation. For instance, aberrant activation of signal transcription factors NF-*κ*B or STAT3, and alterations in p53 status, have each been reported to affect cell survival individually. The presence of the three genes in the same cluster is in agreement with a recent study which examined the hypothesis that alterations in a signal network involving NF-*κ*B, STAT3 and p53 could modulate expression of proapoptotic BAX and antiapoptotic BCL-XL proteins, promoting cell survival [31]. The authors show that over-expression of p53 together with inhibition of NF-*κ*B or STAT3 induced greater increase in the BAX/BCL-XL ratio than modulation of these transcription factors individually. As discussed earlier in this paper, this is a situation in which similar patterns of gene expression are not sufficient to comprehend the biological process. 

In [10], a network depicting Granger interaction among genes from this same gene dataset was presented. The authors analyzed the network in the context of tumor progression and identified gene-gene connections associated with NF-*κ*B, p53, and STAT3. Here, cluster 1 groups not only NF-*κ*B, p53, and STAT3, but also the functionally associated gene BCL-XL, NF-*κ*B regulator A20 and targets IAP and i*κ*B*α*. The presence of NF-*κ*B and fibroblast growth factors (FGFs) and receptors (FGFRs) in the same cluster is also in agreement with the previous work. Members of the FGF family and NF-*κ*B have been shown to interact in various contexts and, despite distinct roles, are involved in cell proliferation, migration and survival [32,33].

Even though MCL-1 and P21 play important roles in cell survival, and BAI1 is transcriptionally regulated by P53, the analysis run here clustered them separately from P53 containing cluster. This result suggests that, in the context of this dataset, their interaction is stronger with genes such as c-JUN, also functionally related to cell survival, proto-oncogene MET and tumor suppressor MASPIN, for instance. Also worth noticing is the interaction of this cluster with the two members of cluster 3: FGF5 and FOP. Like the other members of FGF family grouped in cluster 2, FGF5 is involved in cell survival activities, while FOP was originally discovered as a fusion partner with FGFR1 in oncoproteins that give raise to stem cell myeloproliferative disorders. It would be interesting to identify specific details regarding the intensity and direction of the information flow within this cluster for a clearer understanding of their relationship in the context of cell cycle progression.

## Discussions

Fujita *et al*. [20,21] suggested both a concept of Granger causality for sets of time series and a method for its identification with a statistical test to control the rate of false positives. Although this method is useful for the identification of Granger causality between sets of time series in Bioinformatics and Neuroscience [22], the application was limited to pre-defined clusters, i.e., the time series composing each cluster needed to be previously known. We developed an objective method to define clusters based on the intuitive concept that a gene cluster should interact more intensely in terms of Granger causality within itself than with neighboring clusters.

Krishna *et al*. [34] proposed a Granger causality clustering method based on the structure of a pair-wise network. Their method consists in identifying pairwise Granger causality between gene expression time series and then, by applying the method proposed by Bader and Hogue (2003), to detect dense regions in the network. The difference between their approach and ours is that they take into account the number of edges, and the density of the network which is given by the number of estimated edges divided by the total number of possible edges. The presence of an edge is determined by the p-value’s threshold. Notice that depending on the threshold, the results can change. In our framework, we take into account the weight of Granger causality between sets of time series in order to identify how close two sets are. Consequently, it is possible to obtain a notion of distance between two clusters based on Granger causality, i.e., a continuous measure (distance in terms of Granger causality) instead of a discrete measure (presence or absence of an edge). Moreover, by using the concept of Granger causality between sets of time series proposed by [20], the concept of density of a network can be easily defined in terms of Granger causality instead of a density based on the number of edges as proposed by [34].

A disadvantage of our method is that it cannot be applied for very large datasets. The larger is the number of time series (genes), or the higher the order of the autoregressive process to be analyzed, the higher the chance to generate non-invertible covariance matrices in the calculation of distance (definition 2) and degree (definition 4) between clusters. We believe that this drawback can be overcome through sparse canonical correlation analysis [35], recently proposed in the literature. However, this topic deserves further studies before it can be used in both clustering and identification of Granger causality between sets of time series, since penalized methods relying on L1 penalization [35] or kernel [36] may present biased estimators.

We only analyzed the autoregressive process of order one because gene expression time series data, possibly due to experimental limitations, are typically not large. However, if one is interested in analyzing greater orders, one minus the maximum canonical correlation analysis value among all the tested autoregressive orders can be used as the distance measure between two time series.

The clustering algorithm used here is based on the well-known spectral clustering. Although results were satisfactory, other graph clustering methods may be used. The normalized cuts algorithm proposed by [37], for instance, presents better results in non Gaussian data sets.

Finally, which biological process underlie time series datasets correlation, remains a difficult question to be answered. Studies suggest that correlated genes may belong to common pathways or present the same biological function. However, it is also known that methods based exclusively on correlation cannot reconstruct entire gene networks. Further studies in the field of systems biology might be able to answer this question in the future.

## Conclusions

We propose a time series clustering approach based on Granger causality and a method to determine the number of clusters that best fit the data. This method consists of (1) the definition of degree and distance, usually used in graph theory but now generalized for time series data analysis in terms of Granger causality; (2) a clustering algorithm based on spectral clustering and (3) a criterion to determine the number of clusters. We demonstrate, by simulations, that our approach is consistent even when the number of genes is greater than the time series’ length.

We believe that this approach can be useful to understand how gene expression time series relate to each other, and therefore help in the functional interpretation of data.

## Competing interests

The authors declare that they have no competing interests.

## Authors’ contributions

AF has made substantial contributions to the conception and design of the study, analysis and interpretation of data. KK, AGP and JRS contributed to the analysis and interpretation of mathematical results. PS contributed to the analysis and interpretation of biological data. AF and PS have been involved in drafting of the manuscript. SM directed the work. All authors read approved the final manuscript.
